# Promotion of Cell Migration by Neural Cell Adhesion Molecule (NCAM) Is Enhanced by PSA in a Polysialyltransferase-Specific Manner

**DOI:** 10.1371/journal.pone.0124237

**Published:** 2015-04-17

**Authors:** Feng Guan, Xin Wang, Fa He

**Affiliations:** The Key Laboratory of Carbohydrate Chemistry and Biotechnology, Ministry of Education, School of Biotechnology, Jiangnan University, Wuxi, China; Aix-Marseille University, FRANCE

## Abstract

Neural cell adhesion molecule 140 (NCAM-140) is a glycoprotein and always highly polysialylated in cancer. Functions of polysialic acid (PSA) that binds to N-glycan termini on NCAM remain unclear. ldlD-14 cells, a CHO cell mutant deficient in UDP-Gal 4-epimerase, are useful for structural and functional studies of Gal-containing glycoproteins because their abnormal glycosylation can be converted to normal status by exogenous addition of galactose (Gal). We cloned the genes for NCAM-140 and for polysialyltransferases STX and PST (responsible for PSA synthesis) from normal murine mammary gland epithelial (NMuMG) cells and transfected them into ldlD-14 and human breast cancer cells MCF-7. The effect of PSA on NCAM-mediated cell proliferation, motility, migration and adhesion was studied. We found that NCAM-140 significantly promoted cell proliferation, motility and migration, while polysialylation of NCAM-140 catalyzed by STX, but not by PST, enhanced NCAM-mediated cell migration, but not cell proliferation or motility. In addition, PSA catalyzed by different polysialyltransferases affected the adhesion of NCAM to different extracellular matrix (ECM) components.

## Introduction

The neural cell adhesion molecule (NCAM), a member of the immunoglobulin superfamily, mediates both homophilic (NCAM to NCAM) and heterophilic binding (NCAM to sulfate proteoglycans or other collagens) during cellular interactions[1]. NCAM occurs in three isoforms: NCAM-180, NCAM-140, and NCAM-120. NCAM-140 and NCAM-180 contain a transmembrane and a cytoplasmic region, and are involved in early development and in guidance of migrating neurons. NCAM-120 is linked to the membrane via a glycosylphosphatidylinositol (GPI) anchor, and is up-regulated during differentiation[2,3].

NCAM-mediated cell interactions are modulated by large, negatively charged polysialic acid (PSA)[4,5]. PSA, a linear homopolymer of α2,8-N-acetylneuraminic acid, is typically linked to the fifth immunoglobulin-like domain of NCAM in vertebrates[6]. High levels of PSA are associated with neural development, whereas PSA levels in most adult tissues are low or zero. The presence of PSA modulates the adhesive property of NCAM, and removal of PSA increases NCAM-to-NCAM binding capacity[7]. Polysialylation of NCAM is catalyzed synergistically by two α2,8-polysialyltransferases, ST8Sia II (also called STX) and ST8Sia IV (also called PST), which have 59% amino acid sequence similarity[8].

Overexpression of NCAM and its polysialylated form (PSA-NCAM) have been reported in various metastatic cancers, including neuroblastoma[9], small cell lung carcinoma[10], renal cell carcinomas[11], and Wilms’ tumor[12]. Up-regulation of NCAM expression leads directly to loss of adherens junctions and initiation of tumor invasion[13]. The various pathways are mediated by differential localization of NCAM on the membrane. NCAM-140 localized in lipid rafts activates p59^*Fyn*^ kinase and leads to focal adhesion kinase (FAK) phosphorylation and focal adhesion assembly. NCAM-140 localized in non-raft compartments interacts with fibroblast growth factor receptor (FGFR) through its fibronectin type III domains, and facilitates FGFR-activated signaling, which in turn activates PLCγ and MAPK signaling pathways[13,14]. Enhanced expression of NCAM/PSA-NCAM or of the enzymes PST/STX has been correlated with degree of cancer progression in various studies[15,16]. However, the mechanism whereby PSA is involved in NCAM function remains unclear.

The mutant Chinese hamster ovary (CHO) cell line ldlD-14 is deficient in the enzyme UDP-Gal 4-epimerase. Its abnormal glycosylation can be converted to normal status by exogenous addition of galactose (Gal)[17]. ldlD-14 cells are a useful model system for structural and functional studies of glycoproteins, proteoglycans, and glycolipids[18]. Because the glycan pattern of these cells can be easily manipulated, it is possible to modify the linkage of PSA to NCAM through N-glycans in order to elucidate the role of PSA in NCAM function.

We cloned the *NCAM-140*, *STX*, and *PST* genes from normal murine mammary gland epithelial (NMuMG) cells, and transfected them separately into ldlD-14 and MCF-7 (a mammary cancer cell line) cells. Terminal polysialylation of the N-glycan on NCAM in ldlD-14 cells was controlled by exogenous addition of Gal. Using this experimental system, we evaluated the modulatory role of PSA in NCAM-mediated cell proliferation, motility, adhesion and migration.

## Materials and Methods

### Cell lines and cell culture

ldlD-14, a UDP-Gal 4-epimerase deficient CHO cell line mutant, originally established by Krieger and colleagues[17], was kindly donated by S. Hakomori (The Biomembrane Institute, Seattle, WA), through an agreement with M. Krieger (Massachusetts Institute of Technology, Cambridge, MA). ldlD-14 cells and their transfectants were cultured in Ham's F12 medium (HyClone, Logan, UT) supplemented with 5% FBS (HyClone). The glycosylation status of cells was manipulated by culturing in serum-free Ham's F12 containing ITS (insulin/transferrin/selenium) (BD Biosciences, Bedford, MA) with or without Gal (20 μM). The mammary cancer cell line MCF-7 was from American Type Culture Collection (ATCC; Manassas, VA, USA). Cells were cultured in RPMI 1640 (Hyclone; Logan, UT, USA) containing 10% fetal bovine serum (HyClone), 2 mM L-glutamine, 100 IU/ml penicillin, and 100 μg/ml streptomycin (Gibco; Carlsbad, CA, USA), in a humidified 5% CO_2_ atmosphere at 37°C[19].

### Antibodies and reagents

The antibodies used were mouse anti-NCAM mAb IgG (BD Biosciences; San Jose, CA, USA), mouse anti-β-tubulin I mAb IgG1 (Sigma; St. Louis, MO, USA), anti-His-tag mAb (Beyotime; Haimen, China), mouse anti-PSA-NCAM antibody IgM 5A5 (Developmental Studies Hybridoma Bank, Univ. of Iowa, IA), HRP-conjugated goat anti-mouse IgG and FITC-conjugated goat anti-mouse IgG (Beyotime), FITC-conjugated goat anti-mouse IgM (Biosynthesis Biotechnology, Beijing, China). FITC-conjugated Griffonia simplicifolia lectin II (GSL-II) was from Vector Labs (Burlingame, CA). Fibronectin, laminin, collagen IV, puromycin, hygromycin, DTT, IAM, urea and acetohydrazide were from Sigma-Aldrich (St Louis, MO, USA). Matrigel was from Corning Incorporated Life Sciences (Tewksbury, MA, USA). Other reagents were from Sigma unless described otherwise.

### Gene amplification and transfection

Mouse genes *NCAM-140*, *STX*, and *PST* were amplified by PCR. Primers were designed using the DNAman software program[20] as following: for *NCAM-140*: sense 5'-CCCAAGCTTATGCTGCGAACTAAGGATCT; antisense 5'-CCGCTCGAGTCATGCTTTGCTCTCATTCT; for *STX*: sense 5'-GAAGGCCTGCCACCATGCAGCTGCAGTTCCG; antisense (containing the codons for His 6 residues) 5'-CTAGCTAGCTTAGTGGTGGTGGTGGTGGTGCGTAGCCCCATCACACT; for *PST*: sense 5'-CTAGCTAGCGCCACCATGCGCTCAATTAGAAAACG; antisense (containing the codons for His 6 residues) 5'-GGAATTCTTAGTGGTGGTGGTGGTGGTGCCCTCTGACTGCATGAATAAG. PCR products were digested with the corresponding restriction endonucleases (Takara Bio Inc., Otsu, Japan), and ligated into the vectors pcDNA3.1 (Invitrogen, Carlsbad, CA), pIREShyg3, and pIRESpuro3 (Clontech, Mountain View, CA), respectively. ldlD-14 or MCF-7 cells were stably transfected with the above recombinant vectors using Lipofectamine 2000 reagent (Invitrogen) following the manufacturer's protocol, selected with G418, and designated as ldlD/N140 or MCF7/N140. ldlD/N140 cells stably expressing *STX* gene or *PST* gene were selected with hygromycin or puromycin, respectively, and designated as ldlD/N/S or ldlD/N/P. MCF7/N140 cells overexpressing *STX* gene or *PST* gene were obtained by transient transfection, designated as MCF7/N/S or MCF7/N/P. All the transfections were confirmed by western blotting using anti-NCAM or anti-His-tag antibody.

### Semi-quantitative and quantitative real-time PCR

Total RNA was isolated using a RNApure Tissue Kit (CWBIO; Beijing, China). First-strand cDNA was synthesized using a ReverTra Ace-α Kit (Toyobo; Osaka, Japan). Primers were designed as follows using DNAman software: for mouse *STX*: sense 5’-TCAGAACCAGAACCCAGTCA; antisense 5’-CGACAGTCAGTTTCAAAGCC; for mouse *PST*: sense 5’-ACTGAAAGTGCGAACTGCCT; antisense 5’-GAGAAGACCTGTGCTGGGTC. For mouse *γ-tubulin*: sense 5’-ATCTACCTGTCGGAGCATGG; antisense 5’-GCCTCCCGATCTATGATGTC; Quantitative RT-PCR was performed with UltraSYBR Mixture (CWBIO) using a CFX96 RT-PCR detection system (Bio-Rad). Transcriptional levels of target genes were quantified by the 2^-ΔΔCt^ method[21] and expressed as mean ± SD from triplicate experiments.

### Western blotting

Cells were lysed using T-PER Tissue Protein Extraction Reagent (Thermo Fisher Scientific, Rockford, IL). Protein amounts were measured with a BCA Protein Assay Kit (Beyotime). Equal amounts of proteins were loaded onto 6–10% gradient SDS-PAGE. After membrane transfer, blots were blocked with 5% nonfat milk in PBS for 1 hr at 37°C. Membranes were incubated with specific primary antibodies overnight at 4°C, washed, and stained with respective HRP-conjugated secondary antibodies for 1 hr at room temperature. Signals were detected using a Chemi Doc XRS+ system (Bio-Rad, Hercules, CA).

### Immunofluorescence staining

Cells (2x10^4^) were cultured on 12-mm diameter glass cover slips in 24-well plates for 48 hr, washed with PBS, fixed with 4% fresh paraformaldehyde in PBS, blocked with 1% BSA/0.1% NaN_3_/PBS for 1hr, stained with primary antibody at 4°C overnight, and then with FITC-conjugated secondary antibody for 1 hr at room temperature. Cell nuclei were stained with Hoechst 33342 (Invitrogen, Paisley, UK). Cells were mounted with Glycergel (Dako Cytomation, Carpinteria, CA) and observed by laser confocal fluorescence microscopy (model Eclipse Ti-U; Nikon, Tokyo, Japan) at 600× magnification.

### Motility assay

Cell motility was determined by phagokinetic gold sol assay as described previously[22]. Cells (2x10^3^) in complete culture medium were seeded onto gold sol-coated wells, incubated for 12–18 hr, and photographed under an inverted microscope. Tracking areas of 50 cells were measured using the ToupView imaging system[23] and expressed as square pixels.

### Proliferation (MTT) assay

Cell proliferation was determined by the MTT assay as described previously[24]. Cells (4x10^3^/well) were seeded in 96-well plates and incubated for 12, 24, 48, 72 or 96 hr. Each well was added with 4 μL MTT solution (Cers, China) and incubated at 37°C for 4 hr. The reaction was quenched by addition of 100 μL DMSO, and absorbance at 595 nm was recorded immediately.

### Transwell migration assay

Cells (5x10^4^) were plated in an upper transwell insert (12/24-well transwell; 8 μm polycarbonate membrane; Costar, Corning, NY) in medium containing 0.1 ml of 0.2% BSA (Ruitaibio; Beijing, China). Medium (0.6 ml) supplemented with 5% FBS, serving as a chemo-attractant, was placed in the lower chamber. Cells were incubated for 16 hr at 37°C in 5% CO_2_ atmosphere, washed with PBS, and fixed with cold 4% paraformaldehyde. Cells on the top surface of the insert filter were removed with a cotton wool tip and the filter was stained with crystal violet. Filters were rinsed with deionized water, air-dried, and photographed. Cells in five randomly selected optical fields were counted to determine migration[25,26].

### Flow cytometry

Cells were plated in 24-well plates (2×10^5^ cells/well) in Ham's F12 medium containing 5% FBS for 24 hr, and the medium was replaced by serum-free F12 containing ITS with or without 20 μM Gal. Cells were detached and either (i) incubated with primary antibody at 4°C for 2 hr, then with FITC-conjugated secondary antibody for 1 hr, or (ii) incubated with FITC-conjugated GSL-II at 4°C for 2 hr. Signals from cells were determined by flow cytometry (FACS Calibur, BD, San Jose, CA), with data acquisition and analysis by the FlowJo software program (Tree Star, San Carlos, CA).

### Amidation and separation of sialylated N-linked glycans

Total proteins (2 mg) extracted from ldlD-14/N140 cells were concentrated and desalted using a size-exclusion spin ultrafiltration unit (Amicon Ultra-0.5 10 KD; Millipore, Billerica, MA)[27]. Proteins were denatured with 8 M urea, 10 mM DTT, and 10 mM IAM and centrifuged. Sialic acids were modified using acetohydrazide, as described previously [28,29]. After desalting with water, proteins were digested with PNGase F (New England BioLabs, Ipswich, MA) overnight at 37°C. Released N-glycans were collected and lyophilized. Glycans were desalted using Sepharose 4B (Sigma-Aldrich) as described previously[27].

### Mass Spectrometry

N-glycans were characterized by matrix-assisted laser desorption/ionization time-of-flight tandem mass spectrometry (MALDI-TOF/TOF-MS/MS) (Ultrafle Xtreme; Bruker Daltonics, Bremen, Germany). N-glycans were resuspended in 10 μL of methanol/H_2_O (1:1 v/v) and 1 μL of the mixture was spotted onto the microtiter plate (MTP) AnchorChip (Bruker Daltonics, Bremen, Germany) sample target and air-dried. 1 μL of 20 mg/mL 2,5-dihydroxy-benzoic acid (DHB) in methanol/H_2_O was spotted to recrystallize the glycans. Mass calibration was performed using peptide calibration standards (250 calibration points; Bruker). Distinct N-glycan peaks were obtained from the mass spectra using a signal-to-noise ratio >5 as criterion. Measurements were taken in positive-ion mode, and m/z data were analyzed and annotated using the GlycoWorkbench software program (http://code.google.com/p/glycoworkbench/)[28].

### Cell adhesion assay

Adhesion assays were performed as described previously[30]. In brief, 96-well plates were coated with fibronectin (1 μg/well), collagen IV (1 μg/well), matrigel (40 μg/well) or laminin (1 μg/well) at 37°C for 2 hr. Wells were rinsed and blocked with 1% BSA in HBSS at 37°C for 1 hr. Cells were harvested with trypsin and plated at 40 000 cells per coated well. After 30 min incubation at 37°C, unattached cells were gently removed with HBSS. Adherent cells were fixed with 4% paraformaldehyde for 10 min and stained with 0.1% crystal violet (in 20% MeOH) for 10 min. Excess dye was washed off with PBS, crystal violet in the cells was dissolved in 100 μl 10% acetic acid, and absorbance was measured at 595 nm[31]. Bars represent mean absorbance +/- SEM of each condition tested in triplicates.

### Data analysis

Data were statistically analyzed using the Prism 5 software program[32]. Differences between means were evaluated by Student's t-test, and p-values <0.05 were considered significant.

## Results

### PSA on NCAM is easily modified in ldlD/N140 cells

**Fig 1:**
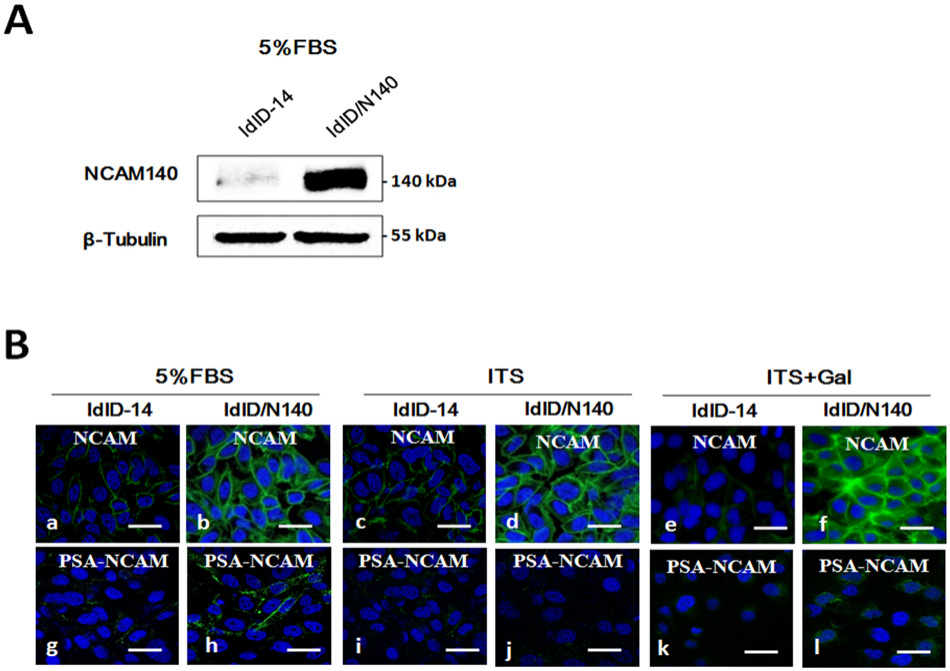
Overexpression of NCAM-140 in ldlD-14 cells. (**A**) Western blot analysis with β-tubulin used as loading control. (**B**) Immunofluorescence staining of NCAM and PSA-NCAM. ldlD-14 and ldlD/N140 cells were cultured in serum-free medium supplemented with 5% FBS or ITS or ITS+Gal, and nuclei were visualized by Hoechst staining. Size bars: 20 μm.

To investigate the function of NCAM-140 and PSA-NCAM, the *NCAM-140* gene from NMuMG cells was cloned. NCAM levels in parental ldlD-14 cells were undetectable, whereas transfected cells (ldlD/N140) showed a strong NCAM-140 signal in all tested conditions (Fig 1A and 1B).

**Fig 2:**
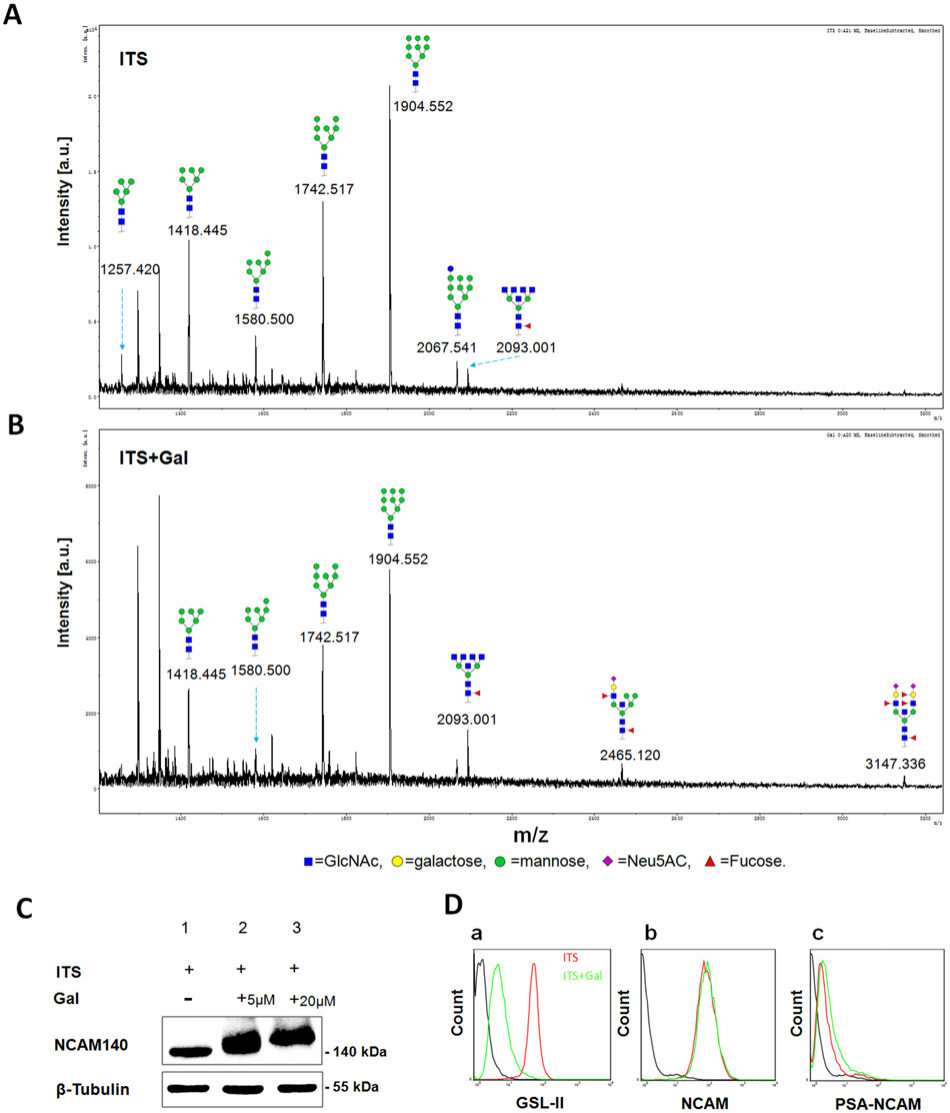
Modification of glycosylation in ldlD/N140 cells. (**A**) and (**B**) MALDI-TOF/TOF-MS spectra of N-glycans in ITS- or ITS+Gal-cultured ldlD/N140 cells. ldlD/N140 cells were cultured in serum-free medium supplemented with ITS (A) or ITS+Gal (B). Derivatized N-Glycans were separated, desalted, and characterized by MALDI-TOF-MS as described in *Materials and Methods*. Representative spectra from triplicate experiments are shown. Detailed glycan structures were analyzed using the GlycoWorkbench program. Proposed structures and their m/z values are shown for each peak. (**C**) Western blot analysis of ldlD/N140 cells cultured in serum-free medium with ITS (lane 1), ITS+5 μM Gal (lane 2), and ITS+20 μM Gal (lane 3). NCAM was analyzed by western blotting with β-tubulin as loading control. HRP-conjugated goat anti-mouse IgG was used as secondary antibody. (**D**) Flow cytometric analysis of glycan patterns using GSL-II (a), anti-NCAM antibody (b), and anti-PSA-NCAM antibody (c).

Modification of glycosylation in ldlD-14 cells by addition of Gal was confirmed by MALDI-TOF/TOF-MS/MS. Since sialic acids are easily lost during mass spectrometric ionization, the glycans released from ldlD/N140 cells cultured with ITS or ITS+Gal were purified, derivatized and analyzed. Proposed N-glycan structures and their molecular weights are shown in Fig 2A and 2B. Cells cultured with ITS had no Gal or sialic acid residues on any of the annotated N-glycans (Fig 2A). In contrast, cells cultured with ITS+Gal presented two unique structures (m/z values 2465.120, 3147.336), annotated as N-glycans with terminal Gal and sialic acid (Fig 2B). This result indicated that ldlD/N140 cells can express NCAM under ITS condition, and express PSA-NCAM under ITS +Gal condition.

In ldlD/N140 cells cultured with ITS, NCAM had a low molecular weight because of the lack of PSA synthesis (Fig 2C). In cells cultured with ITS+Gal, the salvage pathway for Gal incorporation provided an alternative source of UDP-Gal as described previously[33] and the glycan on NCAM was elongated, resulting in a higher molecular weight of NCAM (Fig 2C).

GSL-II, a lectin with N-acetyl glucosamine (GlcNAc)-binding specificity[34], was used to distinguish N-glycan termini of ldlD/N140 cells cultured with ITS vs. ITS+Gal. ITS-cultured cells displayed terminal GlcNAc and a high GSL-II-FITC binding signal, whereas ITS+Gal-cultured cells had almost no fluorescent signal (Fig 2D–2Da). The NCAM pattern was the same under the two culture conditions (Fig 2D–2Db). The PSA-NCAM fluorescent signal was slightly stronger for ITS+Gal-cultured than for ITS-cultured cells (Fig 2D–2Dc).

### NCAM-140 enhances cell motility, proliferation and migration

**Fig 3:**
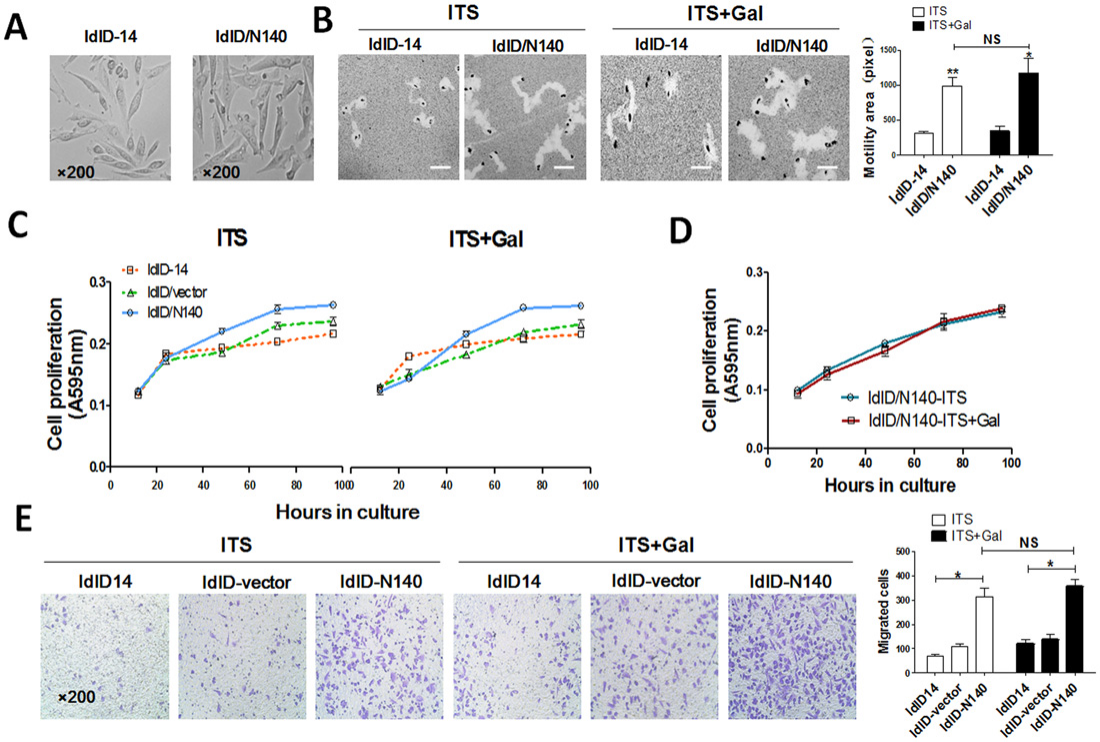
Effects of NCAM-140 on ldlD-14 cell behavior. (**A**) Morphological effects. ldlD/N140 cells were cultured as described in *Materials and Methods*. Magnification: 200 ×. (**B**) Motility assays. ldlD-14 and ldlD/N140 cells were cultured in serum-free medium with ITS or ITS+Gal, and motility assays were performed as described in *Materials and Methods*. Cleared areas on gold sol were measured as square pixels using the ToupView Image program and are shown as mean ± SD from three independent experiments. *, p<0.05; **, p = 0.01–0.05; NS = not significant. (**C**) and (**D**) Proliferation (MTT) assays. ldlD-14, ldlD/N140, and transfected (ldlD-14/vector) cells were seeded in equal numbers (2x10^3^) and cultured in serum-free medium with ITS or ITS+Gal. MTT assays were performed as described in *Materials and Methods*. Values shown are mean ± SD from three independent experiments. (**E**) Migration assays. ldlD-14, ldlD/N140, and ldlD-14/vector cells were cultured for 48 hr as described above. Migration assays were performed as described in *Materials and Methods*. The upper transwell insert contained ITS or ITS+Gal, and the lower chamber was supplemented with 5% FBS. Migrating cells were quantified, and values are shown as mean ± SD. Two independent experiments gave similar results. Magnification: 200 ×. *, p<0.05; NS = not significant.

ldlD/N140 cells, in comparison with ldlD-14 cells, were larger and lost the spindle-like morphology (Fig 3A). They displayed significantly enhanced cell motility (Fig 3B) and proliferation under both ITS and ITS+Gal culture (Fig 3C and 3D). Up-regulation of NCAM-140 resulted in increased migration of both ITS-cultured and ITS+Gal-cultured cells (Fig 3E), suggesting a role of NCAM-140 in metastasis. However, no significant difference of cell motility and migration in ITS-cultured vs. ITS+Gal-cultured cells was observed (Fig 3E, right panel). The proposed reason was that the expression of PSA was not significant enough to make the difference (Fig 1B–1l and Fig 2D–2Dc).

### PSA catalyzed by STX vs. PST has differential effect on NCAM function

**Fig 4:**
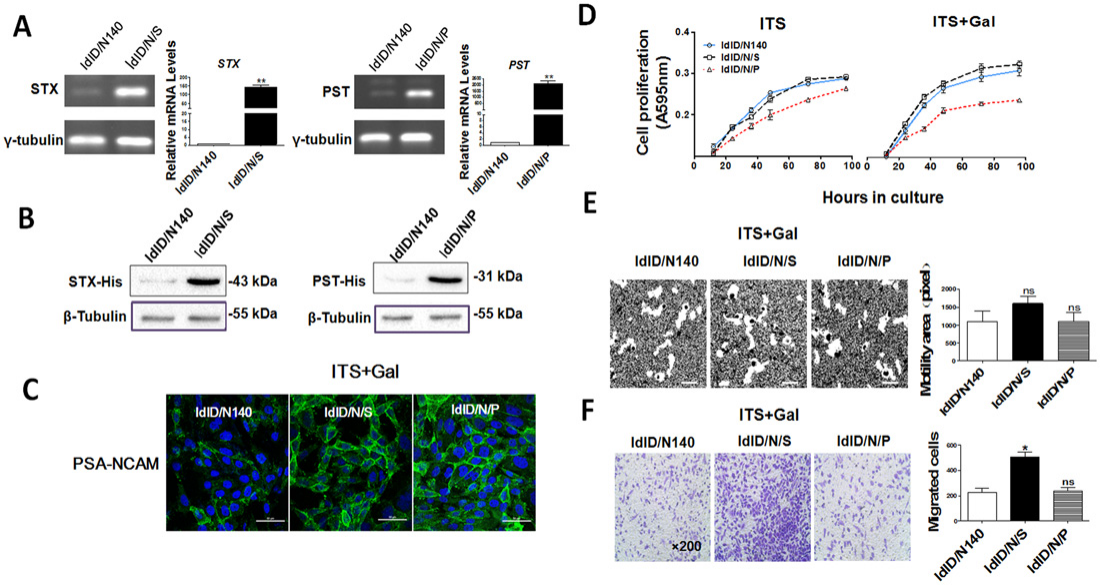
Effects of PSA on ldlD/N140 cell behavior. (**A**) mRNA levels of *STX* and *PST* genes in transfected cell lines were assessed by semi-quantitative (left panels) and quantitative (right panels) RT-PCR, with γ-tubulin as control. **, p = 0.01–0.05. (**B**) Western blot analysis of STX and PST expression using anti-His-tag antibody for selection of transfected cells and β-tubulin as loading control. (**C**) Immunofluorescence staining of PSA-NCAM. ldlD/N140, ldlD/N/S, and ldlD/N/P cells were cultured in serum-free medium with ITS+Gal. Cell nuclei were visualized by Hoechst staining. Size bars: 50 μm. (**D**) Proliferation (MTT) assays. ldlD/N/S, ldlD/N/P, and ldlD/N140 cells were cultured in serum-free medium containing ITS or ITS+Gal, and MTT assays were performed as described in *Materials and Methods*. Values shown are mean ± SD from three independent experiments. (**E**) Motility assays. ldlD/N/S and ldlD/N/P cells were cultured in serum-free medium containing ITS+Gal, and motility assays were performed as described in Fig 3B. Size bars: 10 μm. NS = not significant. (**F**) Migration assays. Cells were cultured and migratory cells were quantified as described in Fig 3E. Two independent experiments gave similar results. Magnification: 200 ×. *, p<0.05; NS = not significant.

The polysialyltransferases STX and PST both catalyze transfer of multiple α2,8-linked sialic acid residues to glycans containing NeuNAc α2→3/6Galβ1→4GlcNAc→R[35]. To directly evaluate the role of PSA in modulating NCAM function, we generated ldlD-14-NCAM140-STX-His (ldlD/N/S) and ldlD-14-NCAM140-PST-His (ldlD/N/P) cells, which overexpressed STX and PST in ldlD/N140 cells, respectively (Fig 4A and 4B). Both of them showed stronger PSA-NCAM signals than did ldlD/N140 cells (Fig 4C).

Proliferation of ldlD/N/S (but not ldlD/N/P) cells was slightly higher than that of ldlD/N140 cells, which had low PSA content (Fig 4D). Cell motility was not affected in ldlD/N/S and ldlD/N/P cells, indicating that NCAM may be the main factor responsible for cell motility (Fig 4E). Interestingly, cell migration was significantly enhanced in ldlD/N/S cells, but not in ldlD/N/P cells (Fig 4F).

**Fig 5:**
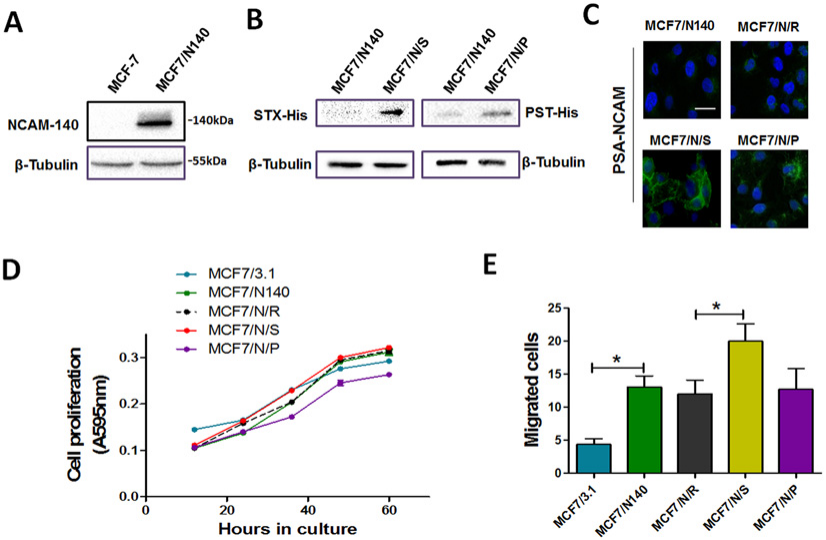
Effects of PSA on MCF-7 cell behavior. (**A**) and (**B**) Western blot analysis of NCAM, STX, PST and PSA-NCAM expression, using anti-NCAM, anti-His-tag antibody respectively and β-tubulin as a loading control. (**C**) Immunofluorescence staining of PSA-NCAM. MCF7/N140 and MCF7/N140 cells transfected with transfection reagent (MCF7/N/R) as mock and control, respectively. Cell nuclei were visualized by Hoechst staining. Size bars: 20 μm. (**D**) Proliferation (MTT) assays. Transfected MCF-7 cells were cultured and MTT assays were performed as described in *Materials and Methods*. MCF7/3.1: MCF-7 transfected with pcDNA3.1; MCF7/N/R: MCF7/N140 cells transfected with transfection reagent. Values shown are mean ± SD from three independent experiments. (**E**) Migration assays. Cells were cultured and migratory cells were quantified as described in Fig 3E. Two independent experiments gave similar results. *, p<0.05.

In order to confirm the above result, a noninvasive breast cancer cell line, MCF-7, was used[36,37]. Stable overexpression of NCAM (MCF7/N140), and transient overexpression of STX or PST (MCF7/N/S and MCF7/N/P) in MCF-7 cells were constructed (Fig 5A and 5B). MCF7/N/S and MCF7/N/P cells showed a strong signal of PSA-NCAM, compared with control cells (Fig 5C). Overexpression of NCAM resulted in slightly increased proliferation, while STX expression, but not PST expression, increased proliferation further in comparison to MCF7/N140 cells (Fig 5D). Similarly, increased migration of MCF7/N140 cells was further enhanced by overexpression of STX, but not PST (Fig 5E). These findings suggested that PSA catalyzed by STX vs. PST has a different effect on cell proliferation and migration, presumably because of a differing degree of polymerization of PSA.

### PSA catalyzed by STX vs. PST has a different effect on the role of NCAM in cell adhesion to different extracellular matrix substrates

**Fig 6:**
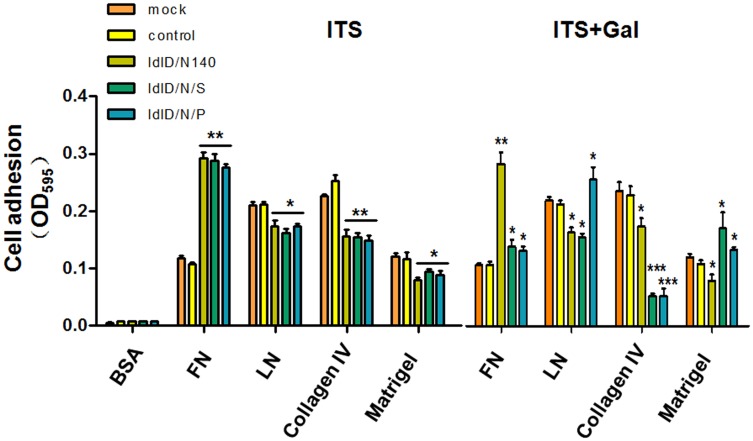
Cell adhesion assay. ldlD-14 (mock), ldlD/plasmid (control), ldlD/N140, ldlD/N/S, ldlD/N/P cells were cultured in 5% FBS medium for 48 hr. Medium was replaced with ITS or ITS+Gal medium and cultured for 48 hr. Cell adhesion to FN, LN, collagen IV, matrigel or BSA solution was determined as described in *Materials and Methods*. The absorbance of fixed and crystal-violet stained cells was recorded at 595 nm. Four independent experiments gave similar results. *, p<0.05; **, p = 0.01–0.05; ***, p<0.01 vs. control.

Literature shows that altered cell adhesion is involved in tumor metastasis and progression, and that the extracellular matrix (ECM) plays an important role in the regulation of cell adhesion[38]. We further examined the effect of NCAM and PSA on the attachment of ldlD-14 cells to the following ECM components: fibronectin (FN), laminin (LN), collagen IV and matrigel (Fig 6). ldlD-14 cells were more adherent to LN and collagen IV than FN and matrigel. In ITS condition, NCAM-140 overexpressing cells (ldlD/N140, ldlD/N/S and ldlD/N/P) were highly adherent to FN and poorly adherent to LN, collagen IV and matrigel, compared with ldlD-14 cells. These different adhesive properties became more pronounced in ITS+Gal condition. ldlD/N/S and ldlD/N/P cells presented decreased adhesion to FN and collagen IV, and increased adhesion to matrigel, compared to ldlD/N140 cells. Interestingly, adhesion to LN was not changed in ldlD/N/S cells, but greatly increased in ldlD/N/P cells. These results suggested that PSA catalyzed by different polysialyltransferases affected the adhesion of NCAM to ECM components.

## Discussion

Cell adhesiveness and migration are key factors in the metastatic process, and modulation of tumor cell adhesion molecules is crucial in control of the metastatic cascade[39]. There is increasing evidence that NCAM functions as a surface marker for human small cell lung cancer[40] and other types of cancer[41]. NCAM function is affected by attached PSA molecules, which are expressed in malignant cancer cells and associated with high metastasis[39].

**Fig 7:**
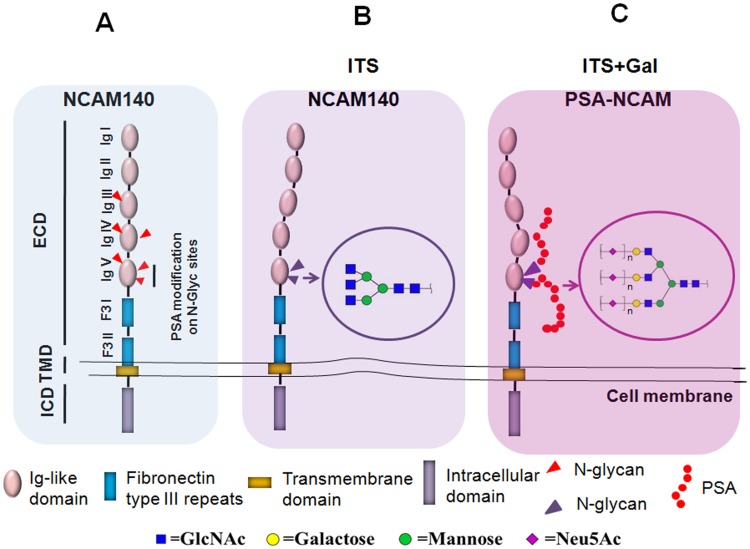
NCAM and PSA expression (schematic) in ldlD/N140 cells cultured in ITS with or without Gal. (**A**) Structure of NCAM-140. The extracellular domain (ECD) of NCAM is composed of five immunoglobulin (Ig)-like domains and two fibronectin type III (F3) repeats. NCAM has six N-glycosylation sites, of which the 5th and 6th have N-glycans that are modified by sialic acid or PSA. TMD, transmembrane domain; ICD, intercellular domain. (**B**) In cells cultured with ITS alone, NCAM-140 has no PSA modification due to the lack of Gal on terminal of deficient N-glycans. (**C**) In cells cultured with ITS+Gal, PSA-NCAM is attached on the NCAM. In the structural diagram, n≥1.

We used an ldlD-14 cell model to elucidate the role of PSA in NCAM function. The UDP-Gal 4-epimerase deficiency characteristic of ldlD-14 cells results in low internal pools of UDP-Gal when cells are grown in the absence of Gal [33]. The derived cell line ldlD-14 is a useful model for studying the functions of glycans in glycoproteins and glycolipids[18,42]. NCAM model in ldlD-14 cells was proposed when cultured in ITS only condition. In this situation, incomplete N-glycan on ldlD-14 cells lacked the factors of Gal for the attachment of PSA (Fig 7B). To study the role of PSA-NCAM, addition of Gal on N-glycan termini is the essential step for PSA synthesis on NCAM (Fig 7C). Using transfected ldlD/N140 cells, we were able to restore normal cell phenotype by addition of Gal and study glycan patterns by flow cytometry and MALDI-TOF/TOF-MS/MS.

In ldlD/N140 cells cultured in serum-free medium supplemented with ITS or ITS+Gal, NCAM-140 strikingly enhanced cell proliferation, motility, and migration without modification of PSA on N-glycan termini. To examine the effect of PSA on NCAM function, we constructed ldlD/N/S and ldlD/N/P cell lines that expressed heavily polysialylated NCAM-140. The polysialyltransferases STX and PST had differing effects on ldlD/N140 cells: PSA catalyzed by STX, but not by PST, significantly enhanced cell migration. Similar results were observed in cancer cells MCF-7.

The hallmarks of tumor malignancy, which refer to invasion and metastasis, frequently coincide with the loss of cell-cell adhesion[43]. High levels of PSA have different effects on cell adhesion to ECM components, such as LN, heparin and matrigel[31]. In our study, cell adhesion to FN was strongly promoted, and cell adhesion to matrigel was reduced by NCAM-140, while these phenomenon was reversed by the presence of PSA. However, the decrease in adhesion to collagen IV by NCAM-140 was further reduced in ldlD/N/S and ldlD/N/P cells. In a previous study, STX and PST differentially directed PSA synthesis in temporal- and spatial-specific manners[44]. STX and PST show differing postnatal expression in various organs and catalyze different length of PSA on NCAM[45]. These properties may account for the differing effects we observed on NCAM function.

In future studies, we will use other cell lines that naturally express NCAM and PSA-NCAM to clarify how PSA enhances metastasis through up-regulation of NCAM, and the differential pathways directed by STX- vs. PST-catalyzed PSA on NCAM.

Some important findings of this study are: (i) ldlD-14 cells provide a useful model for studying and comparing the functions of NCAM and PSA-NCAM; (ii) in our model, up-regulation of NCAM-140 enhanced cell proliferation, motility, and migration, which are characteristic processes of metastatic tumors; (iii) NCAM bearing PSA was associated with enhanced cell proliferation, migration and aberrant adhesion, suggesting that PSA overexpression promotes metastasis; (iv) different polysialyltransferases (STX vs. PST) have differing effects on cell proliferation, adhesion and migration.
